# Development of a three-channel automatic climbing training system for rat rehabilitation after ischemic stroke

**DOI:** 10.1590/1414-431X20208943

**Published:** 2020-06-10

**Authors:** Chi-Chun Chen, Ching-Ping Chang

**Affiliations:** 1 Department of Electronic Engineering, National Chin-Yi University of Technology, Taichung, Taiwan; 2 Department of Medical Research, Chi Mei Medical Center, Tainan, Taiwan

**Keywords:** Ischemic stroke, Rehabilitation, Climbing training system, Rota-rod, Inclined plane, Cerebral infarction volume

## Abstract

This paper reports the development of a three-channel automatic speed-matching climbing training system that could train three rats at the same time for rehabilitation after an ischemic stroke. An infrared (IR) remote sensor was installed at the end of each channel to monitor the real-time position of a climbing rat. This research was carried out in five stages: i) system design; ii) hardware circuit; iii) running speed control; iv) functional testing; and v) verification using an animal model of cerebral stroke. The rehabilitated group significantly outperformed the middle cerebral artery occlusion (MCAo) sedentary group in the rota-rod and inclined plate tests 21 days after a stroke. The rehabilitated group also had a cerebral infarction volume of 28.34±19.4%, far below 56.81±18.12% of the MCAo group 28 days after the stroke, validating the effectiveness of this training platform for stroke rehabilitation. The running speed of the climbing rehabilitation training platform was designed to adapt to the physical conditions of subjects, and overtraining injuries can be completely prevented accordingly.

## Introduction

Stroke is the most common cause of adult disability worldwide. It not only affects patients' behavior, cognition, learning, and memory, but also causes many inconveniences and psychological stresses in daily life, and consequently degrades the quality of life (1). It also results in a heavy financial load to governments, and therefore an issue of immediate concern is to develop an effective rehabilitation mechanism. It has been confirmed that exercise can improve cognitive function in animal models, and can promote neurological recovery after brain injury (2–4). Currently, forced treadmills (5,6) and forced (7) and voluntary running wheels (8) remain the most common training platforms for clinical research and animal model validation. For stroke prevention, the infarction size and the edema caused by middle cerebral artery occlusion (MCAo) can be reduced by at least two weeks of treadmill pre-training (5); subjects benefit more from forced than voluntary exercise due to increased cerebral metabolism, and this observation applies to neuron protection (6). For rehabilitation purposes, voluntary exercise was validated as the most effective invention when upregulating the hippocampal brain-derived neurotrophic factor (BDNF) level and facilitating motor recovery. Forced exercise was the least preferred, and resulted in a high level of psychological stress, a low level of brain BDNF, and poorer motor recovery (8). However, the above-referred training platforms all have their own disadvantages, as explained below.

Compared with spontaneous recovery, treadmill workout has been acknowledged as effective in animal models. For example, before a stroke (9) or after (10), it can result in a smaller cerebral infarct volume or better neurological function. However, as suggested in some studies, low-intensity treadmill training, such as 15 m/min, can benefit animals, while moderate or high intensity training, say 25 m/min, was found to raise the level of serum corticosterone (11). Corticosterone is a typical sign of chronic stress, which usually leads to weight loss and spleen atrophy (12), indicating a negative response to stress. In addition, corticosterone can reduce the availability of BDNF in the hippocampus of a rat (13). BDNF is a neurotrophic factor that affects the synchronic plasticity (14) and can attenuate the neuronal death in many brain injury models (15). Forced wheel running has been experimentally validated to have a neuroprotective effect in a variety of ischemic injury models (5). However, a forced wheel-based rehabilitation at 11 m/min for a 60 min duration every day for two weeks turned out to underperform a voluntary wheel-based counterpart in the recovery from focal ischemia (16). It was concluded that frequent lower intensity exercise, such as provided by voluntary running wheels, is safer for rats with stroke, and has a delayed but sustained effect on BDNF that may support brain remodeling after stroke (16). Although subjects are believed to undergo a lower level of psychological stress when trained on a voluntary wheel (17), they must be screened beforehand to avoid the inter-subject variability (18). Therefore, a median split was used by many to establish an exercise model, or simply excuse underqualified subjects from training.

There are a number of inherent disadvantages in rehabilitation mechanisms using commercial forced animal training platforms. The efficacy of rehabilitation after a stroke has long been a controversial issue, being found by different studies effective (7,10) and ineffective (8,16). From our point of view, such contradiction most likely results from the individual differences and the poor motor function of subjects. For instance, when rehabilitated at a fixed training intensity, physically weaker rats would not only fail to keep up with the running speed of a treadmill, but also would be under severe stress due to electric shock or falling. The worst-case scenario was a rehabilitated group underperforming a non-rehabilitated counterpart (8). In actual clinical practice, stroke severity varies from individual to individual, and hence an adaptive mechanism must be found to optimize rehabilitation efficacy, highlighting the importance of this work even though it is designed for animals at this time.

Over years, our team has been committed to developing novel platforms for rehabilitation purposes (19,20). As a major feature of one study (19), a running wheel was equipped with an acceleration/deceleration mechanism. As soon as a rat exceeded a training threshold, the deceleration mechanism was enabled, and the wheel decelerated at a constant rate. Consequently, the rat got back to the inner bottom of the wheel, and the acceleration mechanism was enabled once an undertraining threshold was reached. In operation, both mechanisms were enabled repeatedly and alternatively, such that the undertraining and particularly the common overtraining problems, say fall injuries, could be resolved simultaneously. In short, a running wheel can be adapted to the physical conditions of a subject using the acceleration/deceleration mechanism.

The level of cortisol has been acknowledged as a marker of psychological stress. As suggested in a recently published paper of ours (21), subjects had an extremely low level of cortisol when rehabilitated using an adaptive training platform compared to those using a forced counterpart, highlighting an advantage of using an adaptive platform. As a matter of fact, the wheel in the other study (20) is an upgraded version of the one mentioned (19). In the first one, the running speed of a wheel simply increased/decreased linearly over time once the acceleration/deceleration mechanisms were enabled, while in the second one, the speed increased/decreased exponentially over time. An acceleration and a deceleration model were fitted to the average raw data of rats collected during a 3-day manual training program. In this regard, the second wheel (20) is a more rat-friendly training platform than the first one (19).

In the present study, a running ladder, or escalator, was designed and used to make three stroke rats climb simultaneously upward as rehabilitation therapy.

## Material and Methods

**Figure 1:**
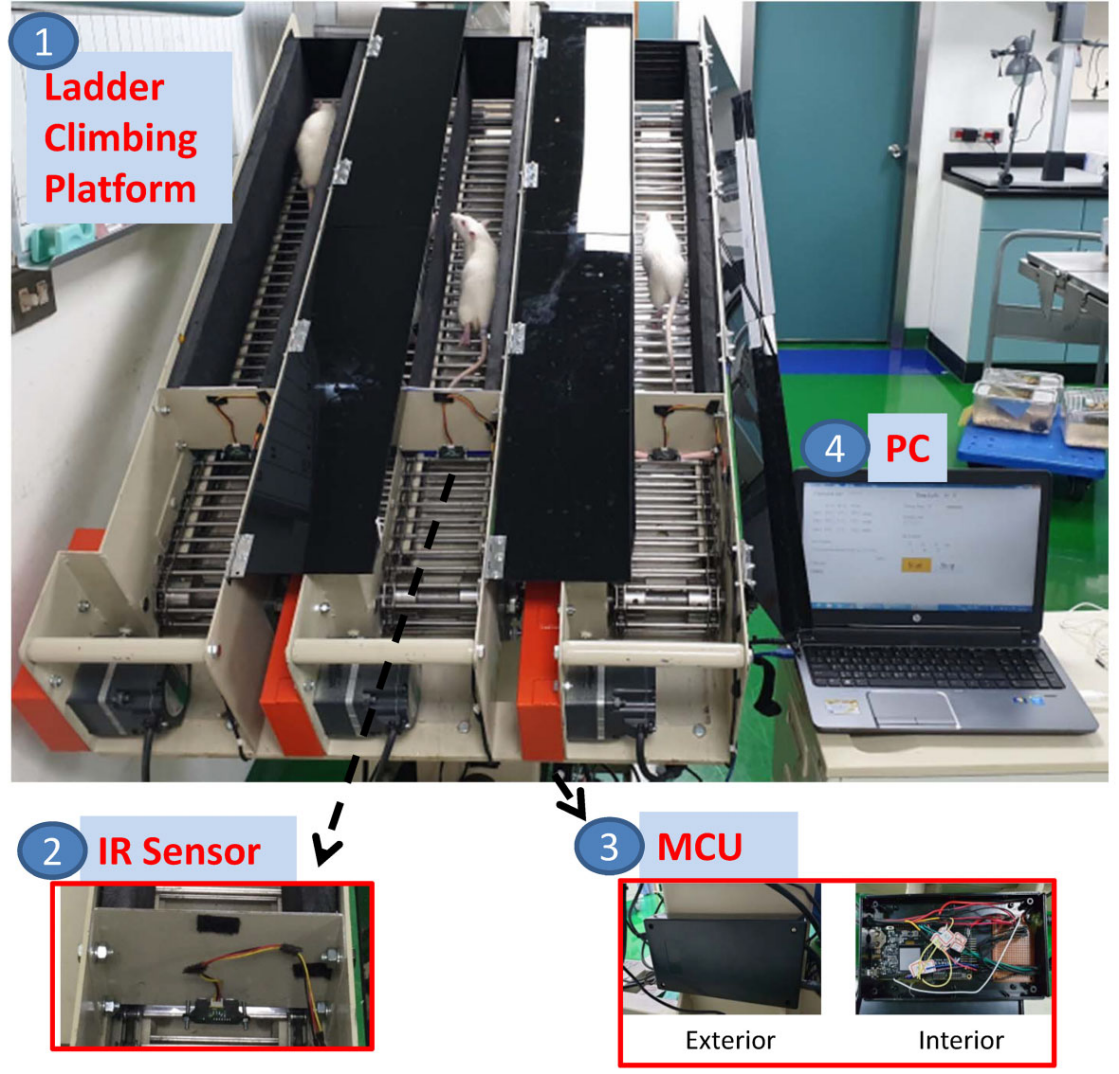
Three-channel automatic speed-matching climbing training system, including 1: ladder body; 2: infrared (IR) distance sensor; 3: microcontroller unit (MCU); and 4: personal computer (PC).

As illustrated in Figure 1, this novel training mechanism consists of four parts: i) a three-channel ladder body; ii) an infrared (IR) distance sensor; iii) a microcontroller unit (MCU) per channel; and iv) a personal computer (PC). The implementation of this training platform involved five stages: i) mechanism design; ii) hardware circuit development; iii) speed control; iv) functional validation; and v) experiments on stroke rats. Stages 1-3 were the development of the platform. Stage 4 was the performance validation, e.g. the position-sensing sensitivity of the platform. Finally, in stage 5, stroke rats were tested on the built platform.

### Mechanical design for the three-channel climbing platform

**Figure 2:**
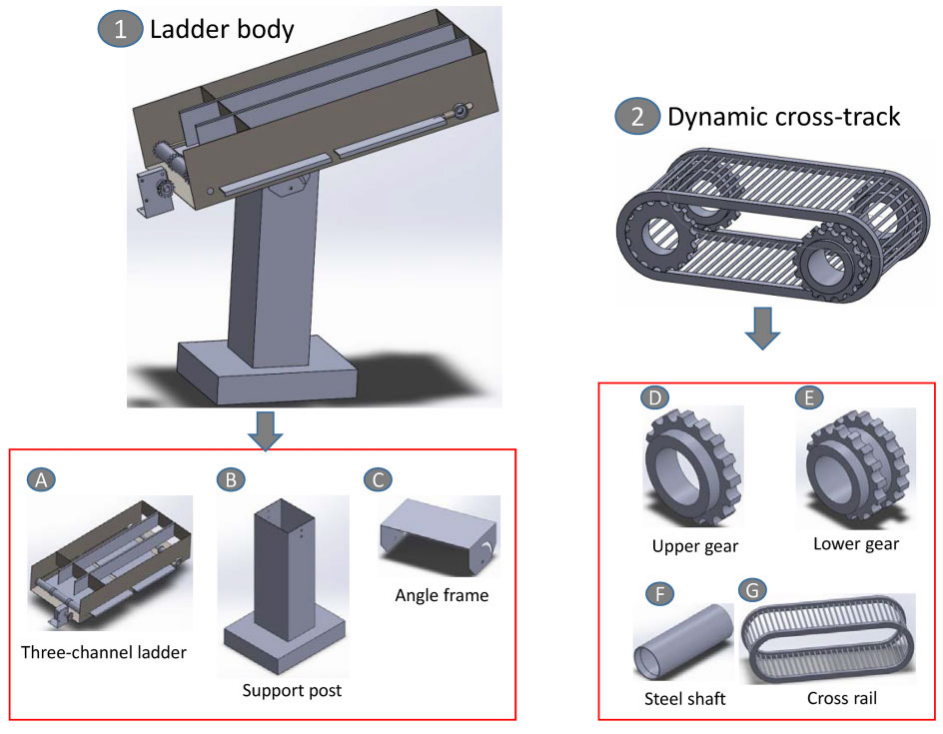
Mechanical design for the climbing platform. The main body of the ladder includes a three-channel ladder (**A**), a support post (**B**), and an angle frame (**C**). The dynamic cross-track includes an upper gear (**D**), a lower gear (**E**), two steel shafts (**F**), and a cross rail (**G**).

The mechanism of the three-channel climbing platform mainly involved a ladder body and 3 identical dynamic cross-tracks. The main body of the ladder consisted of a three-channel ladder, a support post, and an angle frame. The support post and the three-channel base were assembled using an angle frame to form an angle-adjustable system. The dynamic crossbar track was composed of an upper gear, a lower gear, two steel shafts, and a cross rail. The annular cross rail has a pitch of 2 cm, which was hooked to the internal gears of the upper and lower gears. The outer gear of the lower gear was driven by the motor gear, and the upper and lower gears were attached to respective steel shafts. Three dynamic cross tracks were then assembled as the main body of the three-channel ladder (Figure 2).

### Hardware circuit

**Figure 3:**
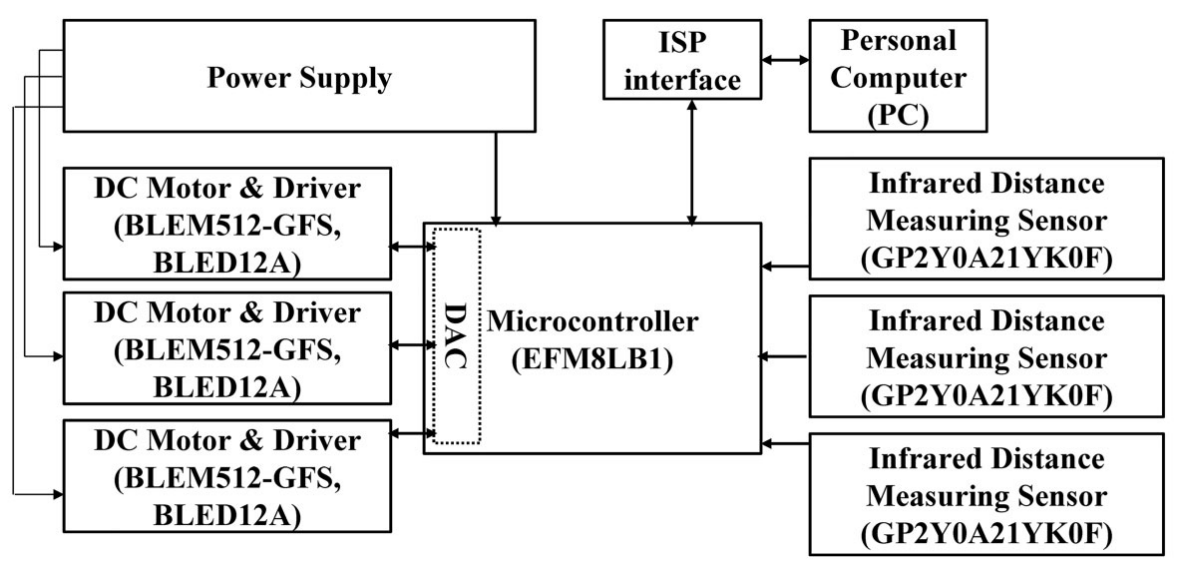
Block diagram of the hardware circuit.

As shown in Figure 3, the hardware circuit was mainly composed of a Silicon Labs EFM8LB1 MCU (USA), three Oriental Motor BLEM512-GFS DC brushless motors (Oriental Motor, Japan), and three GP2Y0A21YK0F distance measuring sensors (SHARP, Japan) to control the ladder running speed and monitor the real-time position of a subject. In each channel, the output of a built-in digital-to-analog converter (DAC) was applied to a direct current motor via an Oriental Motor BLED12A motor driver. Components were configured in such a way that the ladder ran downward. An infrared distance measuring sensor was installed at the end of each channel for position sensing. Sensed analogue signal was then applied to the MCU via a built-in analog-to-digital converter (ADC), and then converted into the real-time position of a rat. Table 1 shows the port assignment for the MCU.

**Table 1:** Microcontroller port assignment.

Port in microcontroller	Function
Port 0.4, 0.5	UART for communicating with PC
Port 1.1, 1.2, 1.3	ADC for receiving IR signals
Port 3.1, 3.2, 3.3	DAC for controlling motor drivers

UART: universal asynchronous receiver-transmitter; PC: personal computer; IR: infrared; ADC: analog-to-digital converter; DAC: analog-to-digital converter.

### Software speed control

A software speed control mechanism aimed to make a rat keep climbing on a 100-cm long ladder. It took rats an average of 30.4 s to climb from the lower to the upper end of the static ladder, that is, an average speed of 2 m/min. The best climber could even reach a maximum instantaneous speed of 7 m/min. With the lower end of the ladder as a reference point and the distance measured in centimeters, this speed control mechanism was designed to rehabilitate rats at 2 m/min and in a midway position (between the interval (41,50 cm)), as much as possible. Table 2 gives the mapping between intervals and assigned speeds of a rat in cycles, formulated as:

**Table 2:** Mapping between intervals and assigned speeds.

Position of the rat by the sensor (cm)	Assigned speed (m/min)
0-10	0
11-20	1.0
21-30	1.5
31-40	2.0
41-50	2.5
51-60	3.0
61-70	3.5
71-80	4.0

P≤X<P+10,P=0, 11, 21, 31, 41, 51, 61, 71 (cm)

Initial velocity=Mapping_table [X]

As can be viewed therein, if a rat climbed faster than 2 m/min and reached the interval (51,60 cm), then the rat was treated as climbing the ladder at a speed of 2.5 m/min at that moment. Instead, when the rat reached the interval (31,40 cm), the rat climbed at 1.5 m/min. As stated in the Introduction, the average speeds of a rat over a cycle were averaged as the initial speed of the ladder, which was then approximated as the nearest speed in Table 2, in the next cycle. The ladder accelerated at 0.1 m/min from the initial speed since the onset of the next cycle, and the instantaneous speed was expressed as

V(t)=0.1t+initial velocity, 0s≤t≤30s

with an upper bound of 7 m/min.

**Figure 4:**
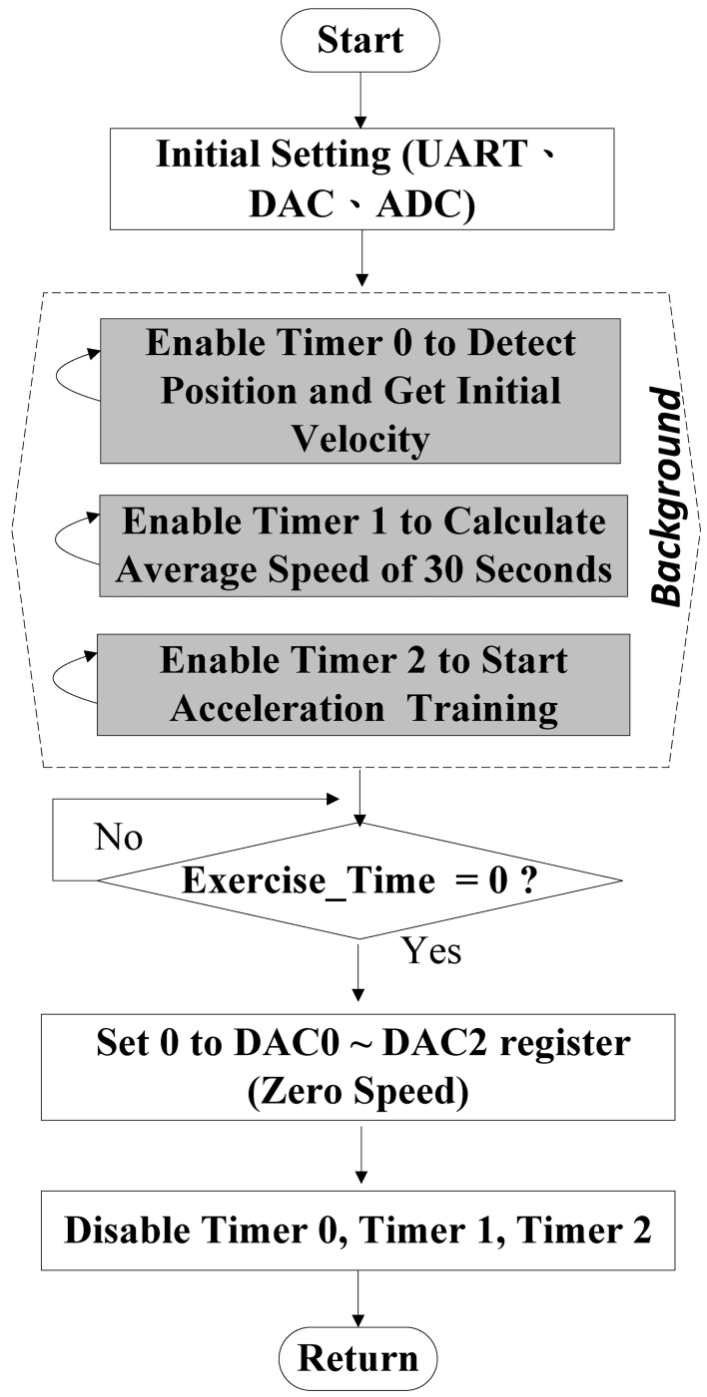
Flow chart of the main software control program of the automatic climbing training.

As illustrated in Figure 4, a universal asynchronous receiver-transmitter (UART) interface was first initialized and then connected to the computer; a DAC was initialized to generate a control signal for the motor in each channel; an ADC was initialized to receive the settings of three IR distance sensor registers. Then, three timer-interrupted service routines were enabled and executed in the background. Timer 0 was used for the position detection so as to assign a speed to a subject over a cycle, as in Table 2; Timer 1 was designed to average instantaneous speeds of a rat over the cycle; Timer 2 was used for the acceleration process. Next, the remaining training duration was indicated by the variable Exercise_Time. When the training terminated, the DAC register that controlled the motors was reset, and the three timer-interrupted service routines were disabled accordingly.

**Figure 5:**
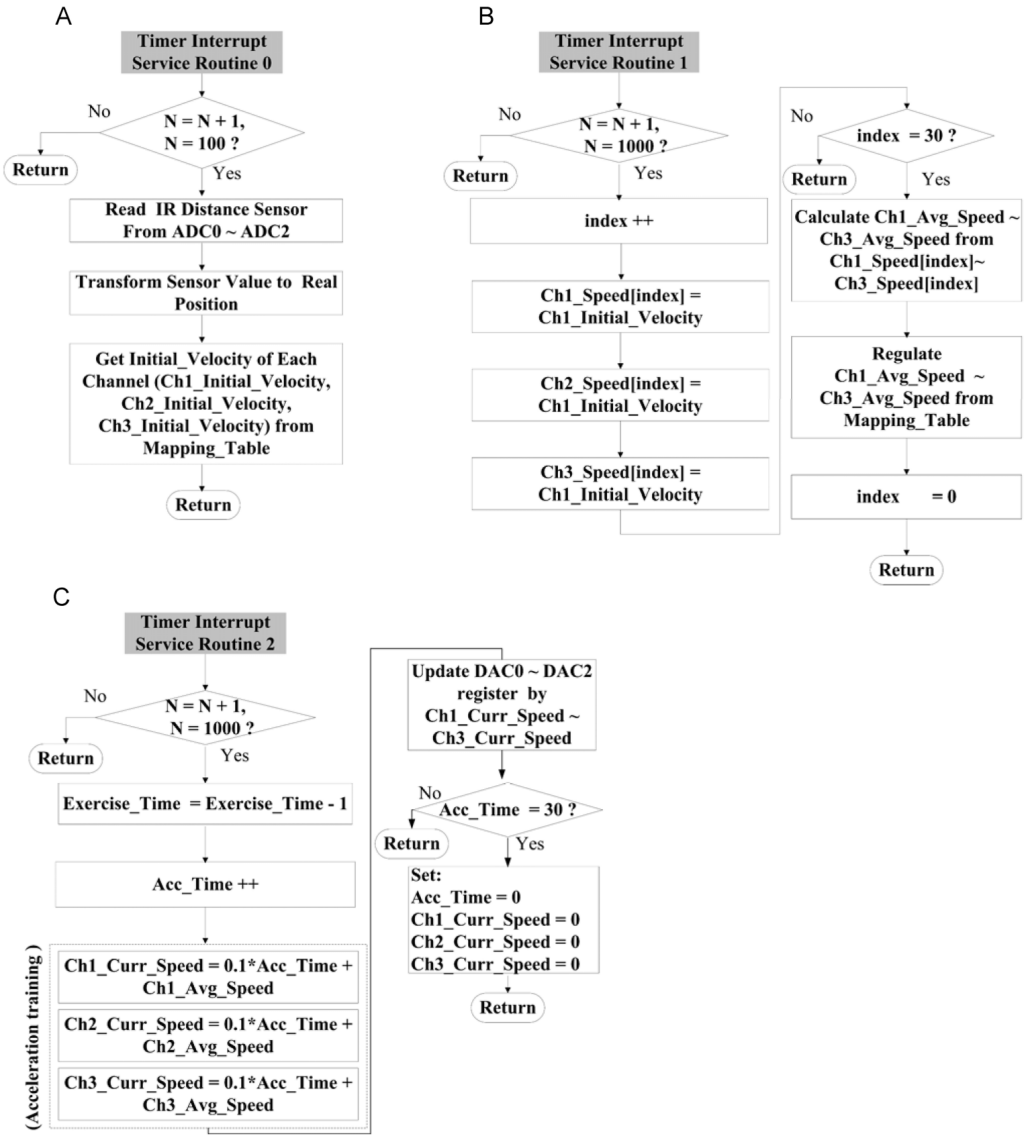
Flow chart of the interrupt service program control software of the automatic climbing training in the Timer. **A**, Operation flow of Timer 0 for position detection. **B**, Operation flow of Timer 1 for 30 s average speed. **C**, Operation flow of Timer 2 for acceleration training.

The operations of timer-interrupted service subroutines 0–2 are illustrated in Figure 5A. The sensed IR signals in each channel were read once per s using timer-interrupted service subroutine 0, and then converted to the current position of a rat using an ADC. Subsequently, as illustrated in Figure 5B, by referencing the mapping in Table 2, an instantaneous speed of the rat was obtained, denoted by Ch1_Initial_Velocity, Ch2_Initial_Velocity, and Ch3_Initial_Velocity. Thirty pieces of speed data over a cycle in each channel, denoted by Ch1_Speed[index], Ch2_Speed[index], and Ch3_Speed[index], were averaged and then denoted by Ch1_Avg_Speed, Ch2_Avg_Speed, and Ch3_Avg_Speed, respectively. Finally, this average speed was compared with the assigned speeds in Table 2, and the closest one was used as the initial speed of the ladder for the next cycle. As illustrated in Figure 5C, the current running speed in each channel, denoted by Ch1_Curr_Speed, Ch2_Curr_Speed, and Ch3_Curr_Speed, was obtained using Eq. 3, and the motor control signal in each channel was generated via the DAC. In this way, the running speed was adapted to the diverse physical conditions of rats.

### System function verification

The position detection accuracy was the key for determining the running speed control of the ladder. In the manual testing, a subject was simulated as a piece of opaque cardboard, to see whether the subject could be accurately located. In the automation testing, the cardboard was glued to the running ladder at low-to-high speeds, that is, 0.1 to 30 m/min. The test results gave a position detection accuracy of up to 98.4%, and met the requirements accordingly.

### Experiment with stroke rats

The goal of this study was to test the effectiveness of rehabilitation training of the prototype in stroke rats.

#### Middle cerebral artery occlusion (MCAo)

The MCAo was performed using the intracranial vascular occlusion method of Longa et al. (22). After anesthetizing the experimental rats, an incision was created from the midline of the neck to the left (0.1 cm). The common carotid artery of the rat was blocked using a string ball, and cerebral ischemia was induced to cause a stroke. After 60 min, the string ball was removed, and the incision was sutured with hemostasis.

#### Animals and grouping

The experimental animals of this study were male Sprague-Dawley rats purchased from BioLASCO Taiwan Co., Ltd. (Taiwan), and weighed approximately 250-350 g. The animals were housed in an air-conditioned room with temperature maintained at 26±0.5°C and light and dark cycles of 12 h each. The rats were free to access drinking water and food. All experiments were carried out during the daytime with lighting. The experimental procedure was approved by the Animal Ethics Committee of the Chi Mei Medical Center, Taiwan.

A total of 32 rats were randomly and equally assigned to four groups: sham, sedentary, treadmill, and ladder groups (8 rats/group). MCAo surgeries were performed in all groups except the sham group, and only the members in the treadmill and the ladder groups were submitted to a protocol. A week after inducing stroke, the rats in the sham and the sedentary groups did not take any exercise training, while those in the exercise training groups (treadmill and ladder) took three weeks of rehabilitation training. The recovery of neuromotor function was assessed using rota-rod and inclined plane tests every week. The rats were sacrificed three weeks later, and the volume of cerebral infarction was obtained using the triphenyl tetrazolium chloride (TTC) assay.

#### Rehabilitation training

**Figure 6:**
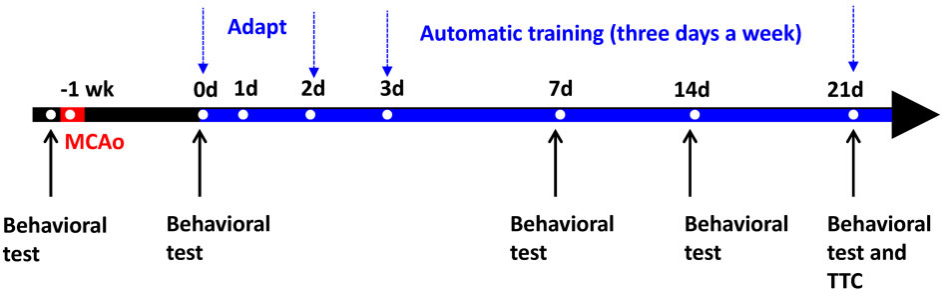
Experimental timeline for verifying the effectiveness of stroke rehabilitation. MCAo: middle cerebral artery occlusion; TTC: triphenyl tetrazolium chloride assay.

The entire training process was divided into two phases: environment adaption and automatic training (Figure 6). All rats received the animal behavior test before the MCAo operation. One week after triggering the strokes, the rats were provided with a two-day adaptive climbing training. The climbing system was stationary on the first day, so that the rats could adapt to the climbing environment and the free climbing process. On the second day, a manual fine-tuning method was used to control the training speed of the climbing, so that the rats could slowly adapt to the speed of the rotation and climb up. If the rat climbed too fast to the position at 70 cm, the ladder speed was adjusted upward to allow the rat to fall back to the position at 40 cm, so it would continue to train near the 40-cm position. On the third day, the automatic training mode started.

Two exercise rehabilitation groups were tested in this study: the treadmill group and the ladder group. For the treadmill group, the training intensity was 10 m/min for 10 min in the first week, followed by a 20-min break, as a cycle, and 3 cycles were performed a day, with a total of 30 min. In the second week, the training intensity was 15 m/min for 10 min, followed by a 20-min break, as a cycle, and 3 cycles were performed a day, with a total of 30 min. In the third week, the training intensity was 20 m/min for 15 min, followed by a 20-min break, and 2 cycles were performed a day, with a total of 30 min (8,10). The training intensity of the climbing group was based on the automatic speed-matching rehabilitation training model, and the training time was the same as that of the treadmill group. The entire training process is shown in Figure 6. There were 3 weeks of rehabilitation training, with three days per week and 30 min per day.

#### Behavioral assessment

**Figure 7:**
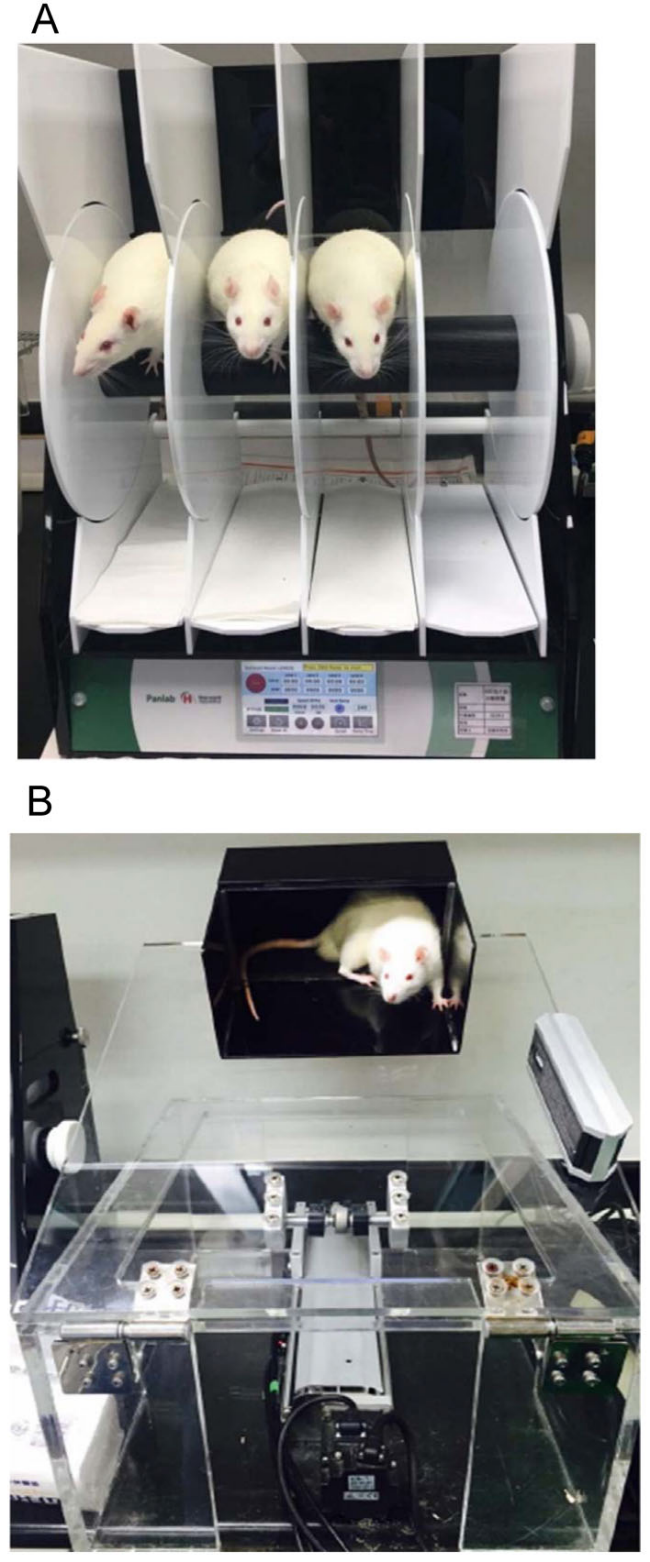
Behavior assessment tools for animal nerve damage. **A**, Rota-rod test (balance bar roller). **B**, Inclined plate test.

A neurological impaired motor function assessment was performed to assess the motor function recovery before and on a weekly basis after the MACo. Two quantitative behavioral assessment tools were used. The first tool was the rota-rod test to assess balance and motion coordination, as shown in Figure 7A (Panlab, Model: LE 8505, USA). A rat was placed on a controllable crossbar at a height of 30 cm above the ground, the initial speed was set to 4 rpm and then stabilized at 30 rpm at 4 min, and the maximum exercise duration was 5 min. During the test, when tired or out of balance, the animal would fall to a weight sensor board, and the moment and the instantaneous speed were recorded. After the first trial, the rat was allowed to rest for 2 min, and the test was performed again. Each rat was tested three times a day (23). The second tool was an inclined plate test to evaluate the grip strength of the hind limbs, as shown in Figure 7B. Firstly, a rat was placed on a rectangular black box, carpeted with a piece of Velcro, at the upper end of an inclined plane. The hind and fore limbs were placed on and outside the black box, respectively. The inclined plane was gradually raised from 25° until the rat failed to catch the Velcro and slid down. At that moment, the plane stopped moving immediately, and the inclination angle was recorded (24).

#### Statistical analysis

Data are reported as means±SD. Data were compared using the repeated-measures analysis of variance (ANOVA), and any two out of the four groups, that is, sham *vs* sedentary, sham *vs* treadmill, sham *vs* ladder climbing, sedentary *vs* treadmill, sedentary *vs* ladder climbing, and treadmill *vs* ladder climbing, were compared using a *t*-test. A difference with P<0.05 was considered statistically significant.

### Results

#### Rota-rod

**Figure 8:**
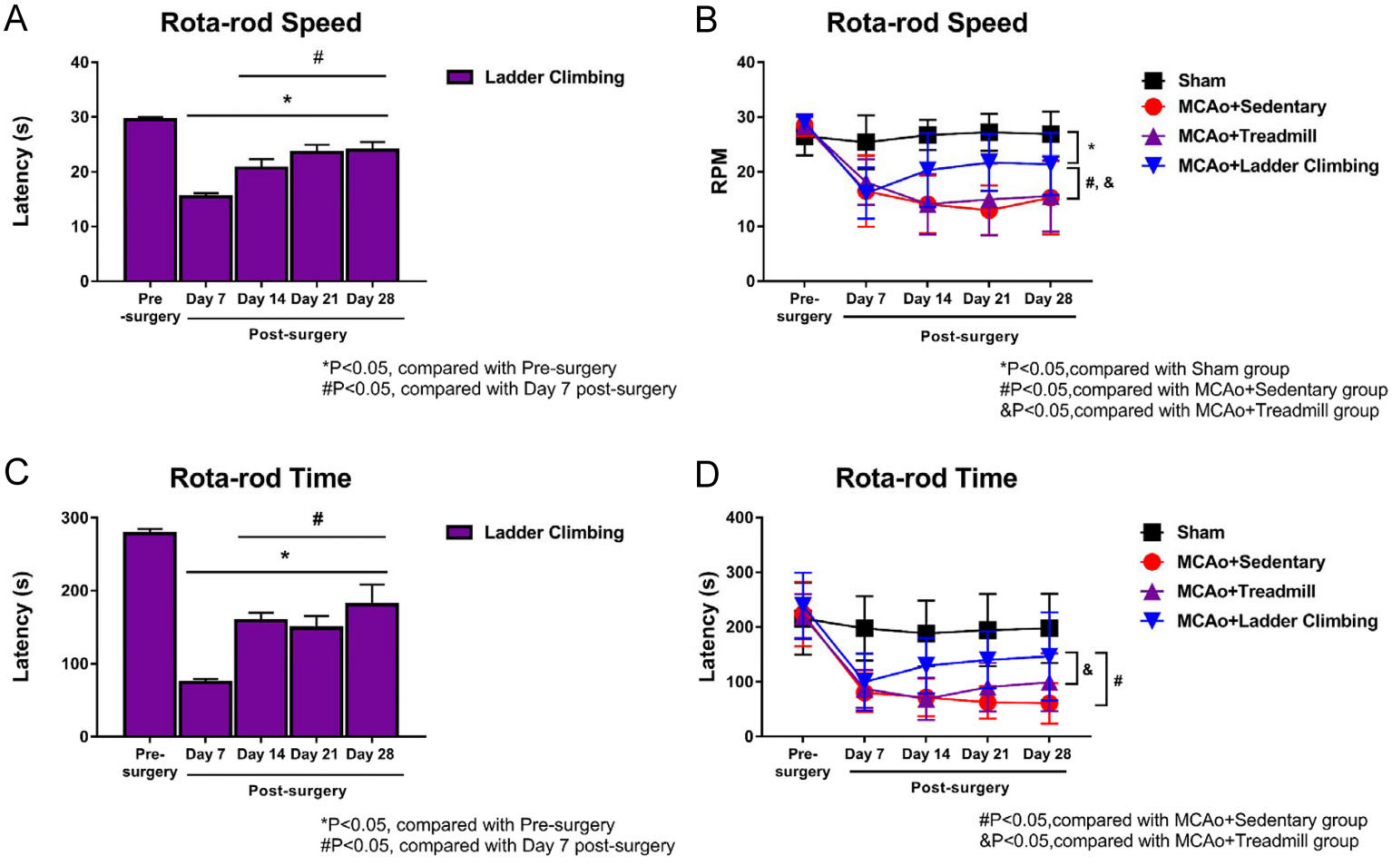
Rota-rod test results for 28 days after stroke rehabilitation in sham rats and rats with middle cerebral artery occlusion (MCAo) submitted to different exercise protocols. **A**, Weekly rota-rod speed for the ladder group. **B**, Average weekly rota-rod speed for each group. **C**, Weekly rota-rod time for the ladder group. **D**, Average weekly rota-rod time for each group. Data are reported as means±SD (ANOVA and *t*-test).

The results of the rota-rod test for the 28 days are shown in Figure 8. Figure 8A shows the weekly speed of the ladder group, demonstrating a significant improvement at the 14th day of rehabilitation compared to the 7th day of rehabilitation. Figure 8B shows the mean speed (RPM) of the weekly rota-rod test for each group within the 28 days, demonstrating no significant difference in the test speed at the 14th day of rehabilitation compared to the Sham group. Figure 8C shows the weekly time of the ladder group, demonstrating a significant improvement at the 14th day of rehabilitation compared to the 7th day of rehabilitation. Figure 8D shows the average latency (s) of each group in the rota-rod test per week, demonstrating no significant difference in the latency of the ladder group at the 14th day of rehabilitation compared to the Sham group. There was also no significant difference between the treadmill group and the sedentary group over the 28 days. These results showed that the proposed ladder training can be effectively applied for the recovery of balance and movement coordination after a stroke.

#### Inclined plane

**Figure 9 f09:**
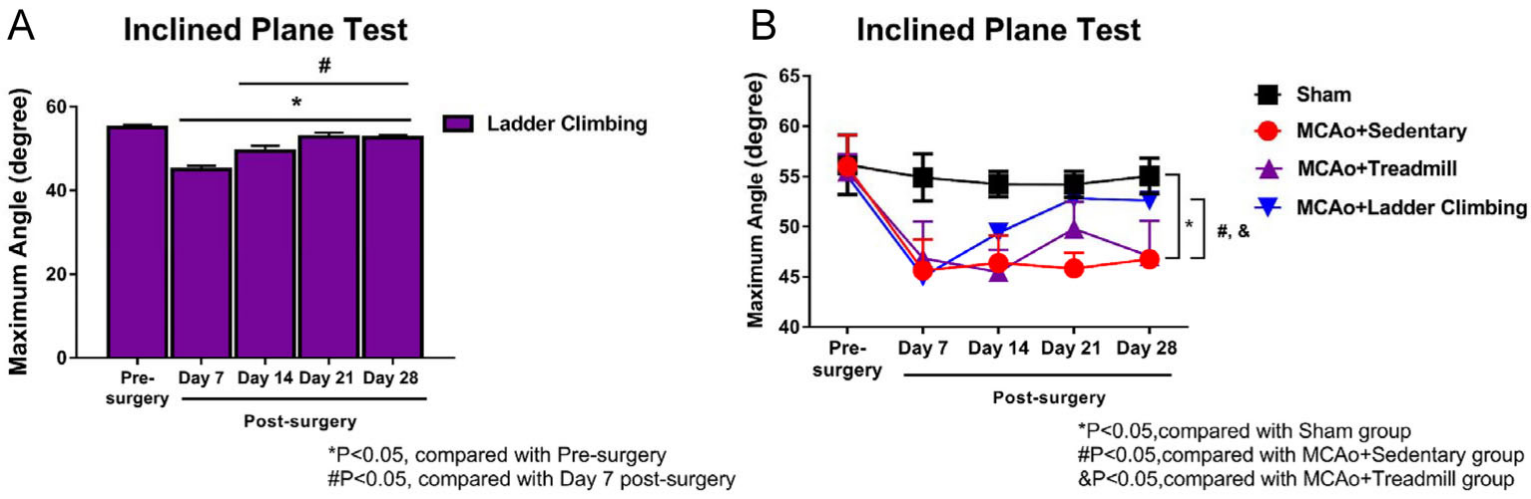
Hind limb grip measured in angles in the weekly inclined plane test for 28 days after stroke rehabilitation in sham rats and rats with middle cerebral artery occlusion (MCAo) submitted to different exercise protocols. **A**, Angles of the inclined plane of the climbing group at each week. **B,** Inclined plane angle of each group at each week. Data are reported as means±SD (ANOVA and *t*-test).

Figure 9A shows the angles of the inclined plane of the ladder group for each week, demonstrating a significant improvement at the 14th day compared to the 7th day. Figure 9B shows the average angle in the weekly inclined plane test for each group over the 28 days. The weekly average inclined plane angle of the sham group was the highest of all of groups, significantly higher than that of the sedentary group, indicating that the MCAo was effective. Figure 9B shows no significant difference between the ladder group and the sham group on the 21st day. The average inclined plane angle of the ladder group on the 14th day was significantly larger from that of the MCAo group, indicating that climbing could significantly restore the hind limb grip ability. There was no significant difference between the treadmill group and the sedentary group within the 28 days. Based on these results, the climbing training proposed in this study can be effectively applied for the recovery of the grasping function in stroke rehabilitation.

#### Cerebral infarction volume

**Figure 10:**
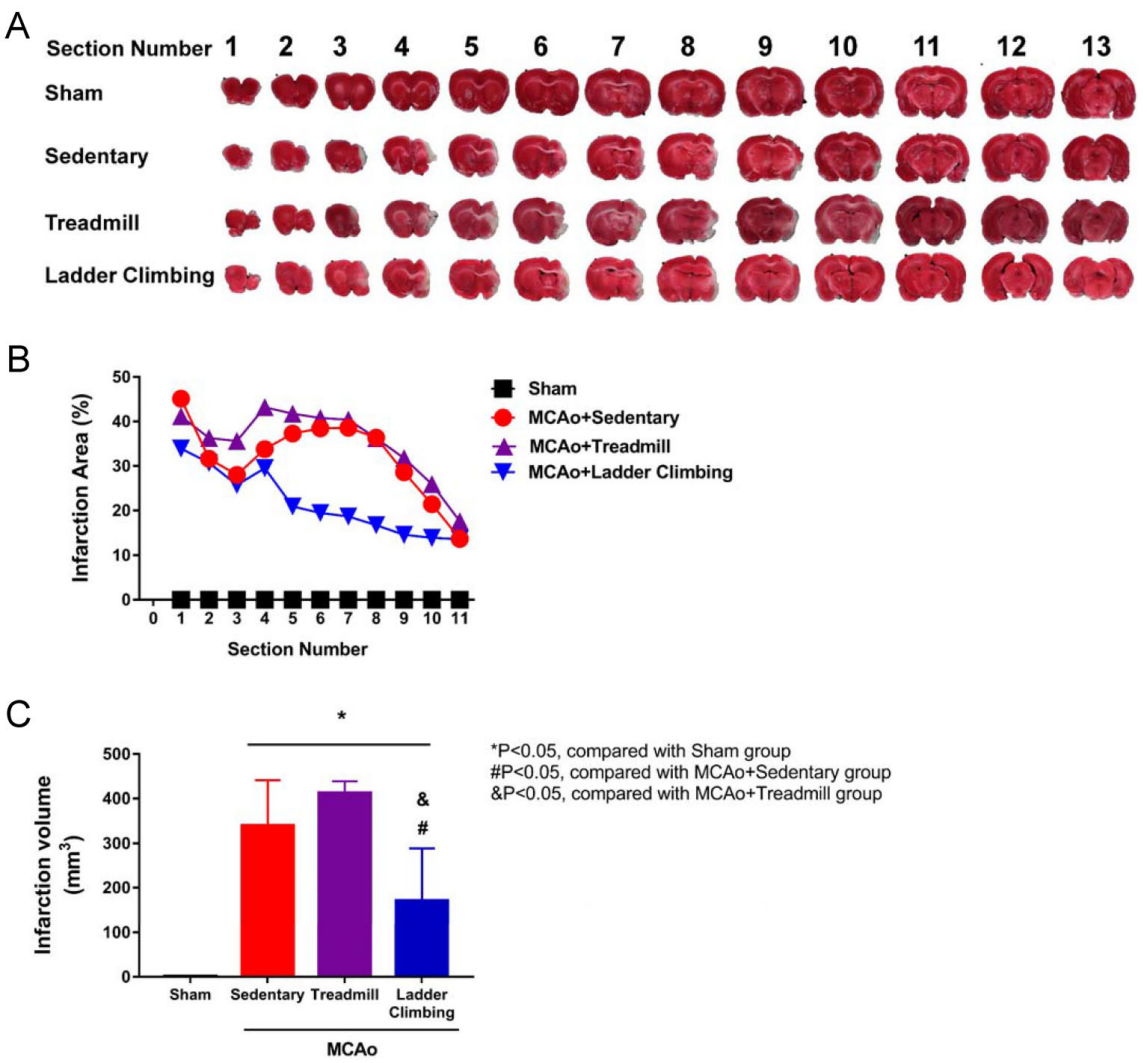
TTC staining to assess the cerebral infarction volume after stroke rehabilitation in sham rats and rats with middle cerebral artery occlusion (MCAo) submitted to different exercise protocols. **A**, Brain section images. **B**, Staining ratio of each brain section. **C**, Cerebral infarction volume on day 28. Data are reported as means±SD (ANOVA and *t*-test).

TTC staining was used to assess the cerebral infarction volume. The normal cells appear red and necrotic cells appear white or eroded, as shown in Figure 10A, which is the staining result of the brain sections from the 1st to 13th sections. Figure 10B shows the percentage of the cerebral infarction volume in each section. There were significant differences in the 5th to 10th sections of the climbing group compared to the sedentary and the treadmill groups. Figure 10C shows a statistical comparison of the volume of necrotic brain cells in each group. The experimental results showed no significant difference in the volume of cerebral infarction between the treadmill group (68.66±5.89%) and the sedentary group (56.81±18.12%), indicating no significant improvement in stroke rehabilitation in the treadmill group. The cerebral infarction volume of the climbing group was 28.34±19.4%, which was significantly lower than that of the sedentary group (P<0.05). Therefore, the climbing training proposed in this study could be effectively used to reduce the volume of cerebral infarction due to a stroke.

## Discussion

As a major feature of this work, 3 rats can be trained at a time, and the training strength can be adjusted according to the physical condition of each subject. A forced animal training platform has been proven to have a positive effect on stroke prevention (5,25), while showing no obvious effects on stroke rehabilitation (8,16). It was suggested that brain damage can be reduced through physical activity before a stroke, attributable to angiogenesis and neurotrophin overexpression in brain regions supplied by the MCA following treadmill workout (25). Spontaneous ladder climbing is a rehabilitation therapy, which is completely different from running on treadmills and running wheels. The position of a rat was monitored in real time and speeds were assigned over a 30 s cycle by referencing a mapping table, and then averaged as the initial speed for the next cycle. Consequently, the exercise was adapted to each condition and resulted in an injury-free platform for rehabilitated rats.

Rehabilitation therapy using a running wheel is not always satisfactory, mainly because stroke rats are physically weak and do not have the same coordination as before a stroke (19). Occasionally, the limbs of a rat are too weak to support its body, causing abrasion between its abdomen and the running belt, which is an inherent disadvantage of treadmill and running wheel-based rehabilitation mechanisms (19,20). The mechanism of the presented rehabilitation is adaptive to the physical conditions of rat subjects, in that overtraining injuries, such as the above-referred abrasion, can be completely prevented. Another major advantage of using this adaptive training platform is a low level of psychological stress due to the absence of an electrical stimulus. Table 3 shows a comparison on the major features among platforms.

**Table 3:** Comparison of major features among animal rehabilitation platforms used in the respective studies.

Features	Treadmill Kinni et al. (6)	Forced wheel running Ploughman et al. (7)	IDRW Chen et al. (19)	Present proposal
Track material	Rubber belt	Cross rail	Rubber belt	Cross rail
Training mode	Forced (electrical stimulation)	Forced (motor-driven)	Adaptive (motor-driven)	Adaptive (motor-driven)
Rehabilitation therapy	Running	Running	Running	Spontaneous Ladder climbing
Number of animals in simultaneous training	Multiple	Single	Single	Multiple
Real-time position detection	Not available	Not available	Available	Available
Automatic speed- matching training	Not available	Not available	Not available	Available
Injury-free training	Not available	Not available	Available	Available
Psychological stress	High	Moderate	Low	Low
Cost	High	Low	Low	Moderate

IDRW: infrared-sensing deceleration running wheel.

The experimental results showed that the motor function and cerebral infarction volume on the 28th day of the rats in the climbing group were significantly different from those in the sedentary group (Figures 8-10), indicating that this climbing system could provide effective rehabilitation training. Therefore, this study demonstrated that a forced training platform can provide a certain rehabilitative effect as long as the training is performed under appropriate control.

The infarction volume difference among groups could be explained as follows. Finger and toe movements had been experimentally validated to stimulate the frontal lobe and corticofugal fibers of stroke patients’ brains (26). From our point of view, this finding could apply to rats, although nothing has yet been reported in the literature to support this argument. In other words, the finger and toe movements of grabbing the railway of a channel under low mental stress conditions are presumed to stimulate and even revitalize respective impaired parts. Accordingly, the ladder climbing feature accounted for considerable reduction in the cerebral infarct volume compared with the treadmill counterpart.

Because climbing does not cause injury due to friction between the body and the instruments and the training speed can be adjusted to match the physical fitness, effective rehabilitation after a stroke can be achieved. The results of the rota-rod and inclined plane tests showed that significant progress was made after the second week of training. Even when the overall average speed of a rat was lower than those of others, it still achieved effective rehabilitation, indicating that different intensities of training should be provided to rats with different physical conditions to achieve maximum efficacy, which can also explain why the traditional fixed-speed training method does not work on all rats.

Psychological stress caused by the electrical stimulation of a treadmill has always been a bottleneck that physiology researchers could not overcome (9). Although the speed of the treadmill in this study was low, most rats could still not achieve good rehabilitation. In addition to the stress of the electrical stimulation, the lack of physical strength and the friction between the body and the track are also reasons for the poor rehabilitation result. Therefore, a training platform that can reduce the psychological burden of training and that is more in line with physical fitness will help neurophysiology researchers to explain experimental data more objectively in basic clinical studies, and the benefits resulting from the exercise will be more convincing. The experimental results proved that a proper training method with controlled intensity can achieve a good rehabilitation effect. Therefore, this rehabilitation model can provide not only an objective experimental motion verification platform for basic clinical researchers but can also be used as a reference for other pathology research topics in the future.

In conclusion, this study proposed a three-channel automatic speed-matching climbing rehabilitation platform that can be effectively applied to the rehabilitation of ischemic stroke in animals. An IR distance sensor provided dynamic feedback that controlled the training intensity, leading to a significant improvement in motor function and cerebral infarction volume, eliminating the electric shock of forced training rehabilitation. This new platform replaces the traditional training method of running with climbing and can also provide a training program according to the physical condition of each individual. The effectiveness of the platform was verified by an animal stroke model.
